# 1009. Case Study. Early-onset Perineoscrotal Hidradenitis Suppurativa Drives Extensive Disease and Complex Reconstruction

**DOI:** 10.1093/jbcr/irag033.197

**Published:** 2026-04-06

**Authors:** Kyung Yoon, Vaisny Balamurali, Kirill Antonov, Tamar Gordis, Michael L Cooper

**Affiliations:** Northwell, Marietta, GA; RUSM, Staten Island, NY; Northwell, Staten Island, NY; Northwell, Staten Island, NY; Staten Island University Hospital Northwell Health, Staten Island, NY

## Abstract

**Patient Presentation (age range, injury details, relevant history):**

Four male patients (ages 18–32) with perineoscrotal hidradenitis suppurativa were treated at a tertiary burn center between 2022–2024. Two had early-onset disease (≤20 years), including one Asian and one African American. Early-onset patients had disease durations of 5–12 years, presenting with multifocal lesions including nodules, sinus tracts, abscesses, and scarring, affecting the scrotum, perineum, groin, buttocks, thighs, and axillae. Later-onset patients had more localized involvement confined to the scrotum and perineum. Comorbidities included diabetes, obesity, and cardiometabolic disorders in two patients, complicating healing. All reported pain, drainage, malodor, and functional limitations.

**Clinical Challenges:**

Early-onset HS created multiple surgical challenges. Extensive tissue involvement across contiguous regions required wide excision with careful attention to critical structures such as the urethra, spermatic cords, and perineal musculature. Complex wound beds with infection, sinus tracts, and chronic inflammation increased the risk of poor graft take and postoperative complications. Early-onset patients often required larger grafts from multiple donor sites, meticulous hemostasis, and staged interventions to minimize morbidity. Additional challenges included managing comorbidities, prolonged hospitalization, and ensuring patient adherence to postoperative care.

**Management Approach:**

All patients underwent a staged surgical strategy. Initial serial debridement with scalpel excision removed diseased tissue while preserving viable structures. Early-onset patients required ≥3 debridements, spaced 3–7 days apart, to allow wound granulation and control contamination; later-onset patients required 2–3 procedures. Definitive closure was achieved with delayed split-thickness skin grafting (STSG) from the thigh or buttock. Grafts were secured with negative pressure dressings or tie-over bolsters, and donor sites dressed with non-adherent dressings. Postoperative care included pain management, infection prophylaxis, wound inspection, and early mobilization. In select early-onset cases, biologic therapy was considered to reduce inflammation and recurrence.

**Outcomes:**

Early-onset patients had longer hospital stays (35–42 days) and one experienced recurrence three years postoperatively. Later-onset patients had shorter stays and no recurrence. All patients achieved definitive wound closure with STSG. Early-onset disease was associated with larger grafts, more complex operative planning, and higher overall surgical burden.

**Lessons Learned:**

Early-onset HS predicts extensive disease and surgical complexity.

Staged excision and delayed STSG optimize outcomes in high-risk patients.

Adjunctive therapies and comorbidity management improve healing and reduce recurrence.

Patient counseling regarding multiple procedures and hospitalization is essential.

**Applicability to Practice:**

Preoperative recognition of early-onset HS allows for anticipatory surgical planning.

Staged reconstruction is effective for complex perineoscrotal wounds.

Multidisciplinary care enhances postoperative outcomes.

Integrating age of onset into clinical decision-making may reduce recurrence and operative morbidity.

